# Veterinary Observations and Remarks on the Diseases of the Horse’s Eye

**Published:** 1818

**Authors:** James Carver

**Affiliations:** Veterinary Surgeon, Philadelphia


### VETERINARY

### OBSERVATONS AND REMARKS

ON THE

### DISEASES OF THE HORSE'S EYE;

Shewing forth the Agency, powerful Influence, and Effect which the effluvia, arising from the Dung and Urine of Stables has, in eausing

### BLINDNESS IN THE EYES OF HORSES:

Being the substance of a Lecture delivered at the

### DUBLIN VETERINARY COLLEGE.

Shewing forth how all these Evils were remedied by the

INTRODUCTION OF VENTILATION,

### BY PROFESSOR COLEMAN,

Through all the Cavalry Barracks of England, Ireland, and Scotland; bringing home conviction to the mind of every unprejudiced man, the impolicy of converting Stables into Store-houses and Manufactories of Manure,

BY JAMES CARVER, VETERINARY SURGEON, PHILADELPHIA;

Interesting to the Physician, the Student, the Gentleman, Farmer and Grazier.

### SUBSTANCE OF THE LECTURE,

INTERLARDED WITH VARIOUS

### OBSERVATIONS, *etc.*

HAVING through all my practice, in this country, both on Long Island and in this city, laid great stress on the necessity of a free ventilation in stables, it will, I hope, sufficiently apologize for being diffuse on this subject.

The great share which litter has in contributing to vitiation of the atmosphere of stables, and in this way producing some, and aggravating many of the diseases of horses, it may be thought there cannot be much necessity for me to go minutely into a detail of its effects under this head. But as I am of opinion that the subject of litter, though not entirely overlooked, has in comparison of its vast importance, been but superficially inquired into, even by scientific writers in our art, I shall, therefore, prefer exposing myself to the charge of tautology in the discussion of it, than omit any point which may appear likely to elucidate the effects of its agency on the health of horses, and thereby probably effect that desirable change so much wanting throughout this country. For I have long been impressed with the conviction, that the materials that are commonly used for litter in this country, are, on account of their being so susceptible of putrefactive fermentation, the chief cause of curtailing the natural period of the life of horses, by silently and slowly undermining the very principles of their frames, and that the volatile alkali engendered in the litter, is, at least, capable of aggravating the disease of cataract, called moon blindness; and the more we consider the phenomena of this disorder, the more we shall be justified in concluding, that the litter is the grand cause of producing this singular affection of the eye. For though it must be admitted that some inexplicable peculiarity of structure does certainly exist in the outer coat of the eye of the horse, which lays him more open to the derangements in its functions than any other animal which we domesticate, yet there can be no question that this prior susceptibility of disease is called into action chiefly by the means of the volatile alkali, which I have long, like Professor Peal, been trying to obtain converts to: I have heard it objected, that dogs that are crowded together in kennels, and which must, consequently, be subjected to the influence of a fouler atmosphere than that of stables, are not particularly subject to disorders of the eyes, and very rarely become blind until old age.

But if this argument be fairly investigated, it will be found rather to strengthen than to invalidate that which I am endeavouring to support. For we must not suppose, that the putrefactive fermentation, the parent of volatile alkali, goes on in kennels, notwithstanding the nauseousness of their stench, in any degree that it does in stables. We must distinguish in fact between the effects of odour, and those of the putrefactive process. The smell of kennels, it is true, is often loathsome to a great degree, from a variety of causes, and chiefly, perhaps, from a concentration of the effluvia which exhales from the skin of dogs, by which means the atmosphere becomes no doubt in a considerable degree vitiated. But it must be recollected that dogs, except in extreme cases, instinctively avoid contaminating their beds with their urine, or fæces, which would be the means of exciting the fermentative process in the straw, and thus, as little or no putrefactive fermentation goes on in their beds, so little or no volatile alkali will be generated from this source. If any one will take the trouble of comparing the pungent effects which are produced upon the eyes and nose, by the air of stables, with the merely nauseous smell produced by that of kennels, he will have no hesitation in admitting the validity of these remarks. Now, the facts that have been already adduced on the authority of Dr. Egan, may serve to convince us, that the urine alone would be capable of diffusing an abundant quantity of volatile alkali in the air of stables, independent of any other source. The professor says, not having an opportunity of making a set of experiments on the urine of different animals which would enable him to speak positively on the subject, he cannot venture to assert, that the urine of the horse contains more urea or gelatine than that of other animals, but I think we may reasonably presume one or other of these circumstances to be the case. For, Fourcroy and Vauquelin have ascertained the difference which exists in different urines, as to the susceptibility of the putrefactive process, depends on the quantity of urea which they contain, and it has been proved that the rapid putrefaction of urine is owing to the action of gelatine on that singular substance. And further, we know from the experiments of these celebrated chemists, that urea is the great source of the volatile alkali, which is generated during the putrefaction of urine, inasmuch as 288 parts of urea were found to yield by distillation 200 parts of carbonate of ammonia, and, according to Dr. Thompson, 100 parts of urea are composed of 39.5 parts of oxygen, 32.5 azote, 14.7 carbon, 13.3 hydrogen. We see then most satisfactorily by the chemical analysis of urea, that this substance contains all the elements of carbonate of ammonia, which is the state, I apprehend, that salt exists in so largely in the air of stables. Now and then, however, it is found in stables in a caustic state; for I have been frequently struck with a fact that takes place when lime washers are employed in whitening the walls of stables, which illustrates the phenomenon called by chemists single elective attraction, and proves at the same time the presence of the salt in immense quantities. For, as the volatile alkali, which is deposited largely on the walls and ceiling of the stable, exists in a state of carbonate, no sooner is the lime applied than the carbonic acid quits the ammonia, to unite itself to the lime, for which it has a greater affinity, and thus at the same instant that the lime is converted into chalk, and becomes mild, by the union of the carbonic acid, the ammonia becomes caustic by the loss of it.

The difference with respect to its pungency is instantly manifest; for I have always observed, that the workmen (especially if they were occupied in lime-washing filthy and ill ventilated stables) become unable to bear the effects of the caustic volatile alkali upon their organs of sight and smell, which, in these cases proves so insufferably pungent, as to oblige them to desist from their work, and run out into the open air, from time to time, in order to escape from its influence. These facts will serve to fortify others that have already been adduced, by way of proving the great quantity of volatile alkali which is mixed with the air of stables; as it cannot be doubted, that such portion of it as is deposited on the walls and ceilings must have its origin from the urine and litter, and has been previously mixed with the atmosphere. In an essay wrote on Farcy and Glanders, by Professor Peal of the Dublin College, also Professor Coleman, has proved that these formidable diseases are the offspring of this ordinary stable management, and that they are more commonly originated by the system of the individuals that are the subjects of them, than propagated, as is usually supposed to be the case, from one horse to another by contagion. And I am strongly impressed with the conviction, that the use of such fermentable materials, as litter, when they came to be acted upon by the urine and fæces of the horse, chiefly give rise to these maladies, which hitherto baffled the utmost efforts of our art. For we can adduce facts to prove, that hot and vitiated air has been found sufficient to excite these diseases in the course of a few hours, in the instance of a troop of horses which were cooped in the hold of a transport, the hatches of which were battened down for three days and three nights: when the hatches were opened on the third day, nine of these horses were found dead, and nearly the whole of the troop were attacked with either farcy or glanders.*

* I have repeatedly heard Professor Coleman say, that if a horse was shut up in a close stable, entirely deprived of ventilation, and the animal

It is a fact well known in some parts of South America, where the heat, not altogether so great, is yet sufficient to expedite the putrefaction of vegetable and animal matter suffered to breathe over and over again the effluvia arising from his own dung and urine, that the glanders would be produced in 72 hours.

Professor Coleman also asserts, that the moment parturition is accomplished, the subsequent existence of the animal depends so much on respiration, that they either enjoy health, activity, and vigour, or become enfeebled, emaciated, and diseased, according to the degree of purity or state of contamination of the atmosphere in which they breathe. For it must be observed, that the air in its passage through the lungs undergoes a decomposition, the oxygen or vital part being absorbed by the blood, and with this part carried to every part of the system, to which it imparts life and vigour; the azotic gas thrown off by expiration, though it may contain its elasticity, is nevertheless deprived of that vivifying principle so essential to life; hence it will appear, that disease must necessarily prevail in stables where a great number of horses stand together, where the roof is low, and there is no ventilation by which the air can ascend. The pavement of these stables also, either from bad execution or want of repair, generally retain the urine evacuated by the horses, which, together with the dung and heat, a noxious exhalation is constantly arising, that greatly tends to increase the impurity of the air.

That abominable custom also, so much in common practice among the grooms of this city, of letting the litter remain under the manger during the whole of the day, merely because it is a little trouble to remove it into the air, should also receive attention.

There cannot be a greater proof of the correctness of this supposition than the following circumstance: When Mr. Coleman received orders from the Cavalry Board of Control, to ascertain and find out the causes of so much blindness existing among the cavalry horses; his first inspection commenced at Woolwich Barracks, where the roofs of the stables were extremely low. The men in ascending the ladders to remove some of the building for the purpose of ventilation, dropped from the ladders like dead rats, which caused Mr. Coleman to believe that the contaminated air which had so long been the cause of producing disease, but more particularly blindness among the horses, was confined in the upper part of the building, which proved to be the case. The roofs having undergone the necessary repairs, the horses were of course returned to their original situation, and I have often heard Mr. C. say, in his lectures, that government, by this salutary step, do not now lose 10 horses a regiment, where they before lost 100.

with great celerity; horses are, on account of their small value, not only not stabled, neither are they subjected to the pernicious effects of fermentable litter, the use of which is, as well as shoeing, comparatively unknown in that part of the world. These are strong, and, I believe, incontrovertible facts. Nevertheless, so impossible is it to extricate altogether, even liberal minds, from the trammels which custom imposes, that I have frequently found it difficult to convince gentlemen that my opinions were not rather the dreams of hypothesis than the result of nearly thirty years' theory, practice, and consideration. And as to people in general in the habit of associating the notion of what is called a comfortable stable, with the conviction of the propriety of the ordinary discipline which grooms and coachmen deem so essential to the well being of their horses, that as I have before observed, that that man who shall propose a free circulation of air, the laying aside the use of clothing, and that horses should stand through the day time upon the pavement, or in their stalls without litter, and all with the sole view of securing them against disease, runs the hazard of being considered a fitter inhabitant for a mad house, than having any pretensions to the possession of his rational faculties. And invariably through all my practice since my arrival in this country have I endeavoured to inculcate the idea, that it is always much better to prevent than to cure disease, and not unfrequently has my opinions been objected to as hypothetical, even from those whose good sense ought to have taught them better,* but contradicted them merely, because it did not suit their convenience to alter them, and that if my arguments were not so, why do we not find, they say, a greater number of instances of moon blindness, farcy and glanders, than we do; inasmuch as the stable system which I decry is pretty nearly the same all over the country. To which I answer, that all glass blowers do not die consumptive; all plumbers do not become paralytic, nor are all chimney-sweepers affect-

* A very strong instance of which occurred in this city in the stable of Wm. Sansom, Esq. of Arch-street, the particulars of which will be noted hereafter.

ed with cancer of the scrotum; but it will be readily admitted by every man who is a competent judge of the facts, that the occupations which these respective persons follow occasions the diseases to which they are peculiarly liable. And though I have repeatedly insisted upon the prejudicial effects of a superabundance of heat in the air of stables, as the means of subjecting horses to rapid vicissitudes of temperature, which occasions so many fatal inflammatory diseases, yet I do not consider that the heat of the air operates to their disadvantage upon the large scale, as the volatile alkali with which it is in a manner saturated, in low confined stables. And this is in fact the sole reason why glandered horses that have been a long while at grass, and appear, on account of the ceasing of the running at the nose, to be cured of the disease, have the disease excited afresh when they are brought back into the stable, although this circumstance is commonly attributed to the heat of the atmosphere of the stable, or to the loss of the green food, which they have been previously accustomed to. For in this instance the volatile alkali operates as a morbid external stimulus to the membrane of the nose, just as it does in that of moon blindness upon the outer membrane of the eye. I have thus glanced at some of the most prominent inimical effects of litter, on the general health of the horse: it now remains for me to speak more particularly of its effects on the feet and legs.

Now, though the facts which Mr. B. Clark has adduced on this subject, must be quite sufficient to satisfy the minds of every unprejudiced person, that contraction of the feet of such horses that stand upon litter, does not proceed faster in the stable, provided they be not shod, than it does while they are at grass; in other words, though this gentleman has proved that iron shoes, be their form or mode of application what they may, is the great source of mischief to the feet; yet I cannot help thinking, that he has somewhat under-rated the influence which the inflammatory agents of the stables must have, in accelerating contraction when once began. For though I am far from doubting the accuracy of my instructor's inductions as to the shoe and nails, being the occasion of most of the evils in the feet, yet I do not think it follows by any means that these instruments are the original cause of the mischief. If, however, it can be proved, that the shod feet of horses that are kept abroad do not contract so rapidly, as the shod feet of those that stand constantly on litter (a fact I think not easily controvertible) it must be pretty plain, that the use of the litter is not only inimical, but one of the chief agents of all the mischief. I am moreover of opinion with what Professor Peal says, that the practice of letting horses stand upon litter is injurious to their feet, for other reasons than merely this of accelerating contraction. For I cannot but consider the soft cushion that the straw furnishes to the feet, must render the elastic fibres that connect the fibres of the coffin bone less capable of bearing the shocks which they are necessarily subjected to, when the horse is trotted or galloped on artificial roads.

Thus it is that horses which are just taken up from grass, and immediately rode upon hard roads, are so frequently attacked with acute inflammation of the lamina, which is a sudden founder of the feet, and so very often supposed to be produced from other causes. The chief means, however, by which litter becomes so prejudicial to the feet of horses, is by heating it; and this it does not by communicating heat to, but by confining it to that organ. For the materials of which litter is composed, being slow conductors of heat, will prevent the hoof from ever becoming so cool and pliable as it otherwise would be, in case the horse stood upon pavement, the materials of which being better conductors of heat than straw, though actually not much cooler, will more rapidly carry off the heat from the hoof.

But he continues to observe, we are entirely misled by our feelings on this important point, and obeying their immediate impulse, our language (philosphically speaking) becomes incorrect. For, if cotton, wool, or fur, which we call very warm articles, be exposed to a frosty atmosphere, and their heat afterwards measured by the thermometer, they will be found to be as cool as marble or stone, that has been exposed to the same temperature, although, if we feel the former, we say, they are warm and comfortable, because they carry off the heat of our skin slowly, and if we touch the latter, we pronounce them extremely cold, because they conduct it very rapidly from the surface of our bodies; it is evident, therefore, that the use of litter must be prejudicial to the feet of horses, by confining their heat, and thereby rendering the hoofs drier, harder, and more liable to crack and chip away, when under the hands of the smith, consequently less elastic, inevitably exasperating the evils which the practice of shoeing brings along with it: the use of oil also, which I have so much reprobated, aggravating the cause, by rendering them incapable of being acted upon when immersed in the bath. And in that disease of the frog called the thrush, which is an inflammation and ulceration of the sensible frog, which lays under the horney insensible frog, wet litter must of course increase the complaint, from the acrimony of the putrid urine, which so often causes a thrush to terminate in canker.

Further, as Mr. Coleman observes that nothing is more inimical to the limbs of horses, or which tends more to cause that winter disease of horses, called grease, which does not proceed, as is supposed, from vitiated blood, or foul humours, but from inflammation of the capillary vessels of the fetlocks, in consequence of the rapid changes of temperature, to which this part of the animal is so frequently exposed; for even if litter is perfectly dry, by surrounding the fetlocks, it will confine the heat of skin, and thus render it very ill calculated to bear the effects of a frosty atmosphere; and what is more, if it be wet, it will act as a two-edged sword, by keeping the skin in an unnatural state of heat so long as the horse continues to stand upon it, and thereby be the means of exposing them to a more intense degree of cold when the horse leaves the stable, than would otherwise follow, in consequence of the rapid evaporation that will take place from the surface, when exposed to the external air; and when once that part is inflamed, the skin cracks and chops, and small ulcers are formed by the acrimony of the putrid urine, which always proves highly irritating, unless we could find some article not so highly fermentable, which might be brought into general use as a substitute for the materials so commonly used on those occasions; and which, by many, may be thought little better than declamation, to inveigh against the mischiefs which they produce. It should therefore be recollected, that there are many evils, both in the natural and moral world, which, though they do not admit of being entirely prevented or remedied, may nevertheless be mitigated in a great degree: this I apprehend to be the case with regard to the use of litter.

For from what has been premised, it must be readily seen, that the mischief resulting from its use are produced not only from one, but from both causes, viz. the heat of the atmosphere and the urine of the animal. But if only a little more attention to cleanliness, and a proper ventilation, which I have so earnestly recommended through all my practice, will enable us to keep the air cool during the hot summer months of the year, how many diseases might be prevented? It should therefore be a great object with every one to prevent the urine from being absorbed by the litter, by every means in our power. For this reason, horses should stand through the day time, upon the pavement, which should be so constructed as to carry off the urine into a reservoir purposely made for its reception, on the outside of the stable. For, though this excretion operates so much to the disadvantage of the health of horses, when it comes to be mixed with the litter, which is of too much value in rural economy, and too precious a material to the practical farmer, not to be husbanded with more care than it appears people in this country have yet any idea of. For although I am writing and endeavouring to convey instruction, which must materially operate against my own practice, it behoves me as a veterinarian to point out those evils which it occasions in producing disease, and I also consider it a duty not less incumbent upon me, to advert to the importance in which this article, as a manure, is now considered all over Europe. In fine, the urine of the horse is now considered to be of more value than the dung itself, so much so, that this subject has of late excited the attention of many of our first chemical philosophers, and we may rest assured that we are but yet in our infancy of knowing rightly how to appreciate its value, as applicable to the purposes of agriculture. For it is the very property of urine which occasions it to be so very hurtful to the health of horses kept in stables, which renders it of such valuable importance as a manure, namely, that of being so readily susceptible of putrefactive fermentation; the farmer ought, therefore, to preserve it with as much care as the apple of his eye. The contents of the reservoir should, from time to time, be thrown upon such heaps of dead vegetable matter as he wishes to convert into manure by the putrefactive process, and in order to save and accumulate the greatest quantity possible, the pavement of the stable should be so constructed, and of such materials as to admit of very few interstices, for they occasion the lodgment of the urine, which readily gives the first impulse to the fermentative process in the litter, especially when assisted by the heat communicated to it from the body of the animal when he lays down. Wooden floors, though in general use in this country, are not good for many reasons: in the first place, they are seldom or ever made as they should be, of the very heart of well seasoned oak; and wood in any shape, even if constructed of the very best materials, will imbibe and absorb the urine, from which must, even with all our care, exhale volatile alkali.

The odds and ends of the pieces of stone which we see lying about the stone-cutters' yards, I have often thought would be an excellent material for this purpose, and the stall might always be so constructed as to admit of a small slope on each side of it, and by having a small iron grating placed in the middle, which might always be so constructed as to be removed at pleasure for the admission of the urine, would, I think, be a good material for this purpose. And for reasons which have been before stated, it must be evident that the pavement of stables, when constructed of convex stones, as they generally used to be in Europe, must always contain a quantity of leaven, if I may so speak, sufficient to communicate the putrefactive process to the fresh litter when laid over it.

And for this reason, it would greatly contribute to sweeten and cool the atmosphere of stables, if gentlemen would make their servants, during the summer months, wash their stalls once a day; and this might always be done so as not to dilute the urine in the reservoir. The whole of the litter should be removed early in the morning, and the wet parts carefully selected from the rest, in order to be thrown upon the dung-hill; the remainder, whenever the weather would permit, should be spread abroad for some hours in the sun and open air, suffering no heaps of litter or fæces to remain in or near the stable.

This plan of managing litter, to be sure, would be attended with a little more trouble to Tom, Dick, and Harry; however, be that as it may, it would not only contribute to purify the air, but to the general health of the horse.

The mere saving of straw, however, would be a bad species of economy to every man whom manure was an object to, particularly if the utmost care was taken to prevent the urine running to waste, but should, from time to time, be thrown on the dung heap, which would be the means of saturating the whole of the straw with the urine.

And though this method of managing the straw, when the saving of it becomes an object, by applying the urine to it out of the stable, will be found to render the mass of manure less in quantity than what is produced by the usual mode, yet the quality of it would be materially improved, and the farmer might use it in the state of what is called long dung. For, the equable application of the urine to the straw, will insure the rapid putrefaction of the entire mass. And as the use of long dung, on the principle of economy, has been strongly recommended by that illustrious chemist, Sir H. Davy, and is approved of by that celebrated Norfolk farmer, Mr. Coke, and many other enlightened practical agriculturists, solely on this account, it must therefore be pretty evident to the advocates of this system of manuring, that the method of saving and applying it to the straw, which I have recommended, holds forth incalculable advantages. For, as long as urine and straw can be obtained, the process of manure-making may be constantly and regularly carried on. And if any urine should remain, after the straw is completely saturated, it might be advantageously employed in forwarding putrefaction, in any other sort of dead vegetable matter, and thus considerably contribute to the interests of the farmer. Indeed the circumstances of saving straw would, to many, prove a loss to them, as it is by no means an uncommon practice, in some places, to supply the livery stable-keepers with straw for the sake of receiving the manure in return.

But all proprietors of horses will find it more to their advantage to encourage the putrefactive fermentation of the litter out of doors, than in their stables; which, judging from the pungency of the atmosphere, and the heat of too many, one would imagine was the object meant to be obtained. At those times, and at those places however, where the value of straw becomes great as food for cattle, the dried haulm or potatoe tops may be substituted for it as litter, and has been recommended for that purpose, on the highly respectable authority of Mr. Curwen: but though there can be no doubt that the haulm would be a very good substitute for straw, yet the use of it for litter would require great circumspection as to the cleanliness and ventilation of stables; for, as it contains a great deal more fermentable matter than straw, so there can be no doubt that it would be the means of diffusing more volatile alkali in the atmosphere. Many individuals, particularly in this country, and I believe in all parts of it, might readily find a supply of saw dust, which would make an excellent litter, especially that produced from all species of firs; also the dried leaves in the woods, which would resist putrefaction very strongly, on account of the turpentine they contain.

In many parts of Europe, but more particularly in India, sand is used as a litter to lie on.* Sand and saw dust I know is good, and in this country might be readily procured: however, the inimical effects which litter has upon the health of horses, when composed of the ordinary materials, and managed as they now are in this country, it must nevertheless be admitted that a considerable portion of good springs to the community from this very evil. For, though the superiority of stable manure over many other kinds of putrid vegetable, and animal composts, has been considerably overrated, yet it cannot be denied that it is of immense value in agriculture.

The grand object however with me, in writing this Essay, is to point out and expose whatever I may have seen and witnessed, particularly on Long Island and among the Pennsylvania farmers, as well as in the city of Philadelphia, what I know to be so productive of causing so much blindness, as well as every way prejudicial to the general health of

* As respects litter, I cannot help making some remarks here respecting this article, which will no doubt both surprise and astonish many respecting the use which is made of the dung of horses and elephants for litter, but more particularly among the princes, nabobs, and great men of the East, and which they prepare in the following manner:—The dung is dried in the sun, and sometimes pounded and sifted, and then laid in the stall about a foot or more in thickness for the horse to lie on, and the glossy appearance and effect this material has on the skin of the animal is surprising. The dust on the animal is removed in the morning by a cloth, and he is then well rubbed with thick coarse hair mittins. Tan, if it could once be properly introduced, would, I should suppose, make one of the finest substitutes for litter in the world.

that noble animal. For, on Long Island, it is notorious, that three horses out of every five through the island, is blind with cataract; and if any one will only take the trouble for the space of one week, to walk up and down Market-street, he will, I believe, find that I am not very far out of my calculation, as respects this state and city. This certainly is, or at least ought to be, a paramount consideration with those whom it may concern; though perhaps, if we look at it in another light, there is no one more concerned than myself; for if people are determined to continue on, in their old way of stable deception, by suffering their horses to stand literally on hot beds of manure, and are determined to give no ventilation to their stables, certainly, as a veterinary practitioner, the more grist will continually be coming to my mill; whereas, by bringing home to every man's bosom these facts, which, in the course of time, must, and will be brought on, I shall, of course, never die a nabob. I shall now close this subject, by recommending the most scrupulous preservation of urine in reservoirs, instead of suffering it to be diffused in puddles in the manner it now is in stables generally, and thereby suffered to run to waste.

I have endeavoured in fact to show, not only that urine becomes the great means of heating and vitiating the air of stables, and thereby laying the foundation of diseases, but that this process, so necessary to the farmer, may be as effectually carried on, as far as the object of manure is concerned, out of doors, though perhaps not quite so rapidly as in the stable. And as facts, not theory, have long ago proved to many of our first veterinarians, the inestimable value of urine as an article of manure, I have, with them, laid considerable stress upon the necessity of carrying it off into reservoirs, for the purpose of afterwards being thrown, from time to time, upon such materials as require to be fermented.

For however strong my conviction may be of the impolicy of converting stables into storehouses and factories of manure, I might have hesitated in urging the matter so strongly, if I had not seen that the industrious farmer might be benefited, as well as the general health of the horse secured against disease, by attention to these few hints loosely thrown together, and after what has now been said on this important subject, it will not, I hope, be thought superfluous to suggest, that dunghills should never be made in situations where the unwholesome vapours arising from them can find their way into the stable.

J. C. V. S.

